# COVID-19 Patterns in Araraquara, Brazil: A Multimodal Analysis

**DOI:** 10.3390/ijerph20064740

**Published:** 2023-03-08

**Authors:** Dunfrey Pires Aragão, Andouglas Gonçalves da Silva Junior, Adriano Mondini, Cosimo Distante, Luiz Marcos Garcia Gonçalves

**Affiliations:** 1 Pós-Graduação em Engenharia Elétrica e de Computação, Universidade Federal do Rio Grande do Norte, Av. Salgado Filho, 3000, Lagoa Nova, Natal 59078-970, Brazil; 2 Institute of Applied Sciences and Intelligent Systems-CNR, Via Monteroni sn, 73100 Lecce, Italy; 3 Instituto Federal do Rio Grande do Norte, Rua Dr. Mauro Duarte, S/N, José Clóvis, Parelhas 59360-000, Brazil; 4 Faculdade de Ciências Farmacêuticas, Universidade Estadual Paulista “Júlio de Mesquita Filho”, Rodovia Araraquara-Jaú, Km 1, Campus Ville, Araraquara 14800-903, Brazil

**Keywords:** COVID-19 dynamics, social distance, lockdown, time-series forecast

## Abstract

The epidemiology of COVID-19 presented major shifts during the pandemic period. Factors such as the most common symptoms and severity of infection, the circulation of different variants, the preparedness of health services, and control efforts based on pharmaceutical and non-pharmaceutical interventions played important roles in the disease incidence. The constant evolution and changes require the continuous mapping and assessing of epidemiological features based on time-series forecasting. Nonetheless, it is necessary to identify the events, patterns, and actions that were potential factors that affected daily COVID-19 cases. In this work, we analyzed several databases, including information on social mobility, epidemiological reports, and mass population testing, to identify patterns of reported cases and events that may indicate changes in COVID-19 behavior in the city of Araraquara, Brazil. In our analysis, we used a mathematical approach with the fast Fourier transform (FFT) to map possible events and machine learning model approaches such as Seasonal Auto-regressive Integrated Moving Average (ARIMA) and neural networks (NNs) for data interpretation and temporal prospecting. Our results showed a root-mean-square error (RMSE) of about 5 (more precisely, a 4.55 error over 71 cases for 20 March 2021 and a 5.57 error over 106 cases for 3 June 2021). These results demonstrated that FFT is a useful tool for supporting the development of the best prevention and control measures for COVID-19.

## 1. Introduction

*Sarbecovirus* SARS-CoV-2, the causative agent of the coronavirus disease known as COVID-19, was responsible for one of the most important health emergencies in the world. Infection with SARS-CoV-2 can be asymptomatic, present mild disease, or lead to upper respiratory symptoms and extrapulmonary syndromes that can cause death [1].

The virus spread occurs mainly through airborne transmission and contact with droplets and fomites [2,3]. In the absence of vaccines or other pharmaceutical interventions, the first control measures to avoid a growth in incidences were the use of masks and a reduction in community mobility. These non-pharmaceutical interventions (NPIs) were the initial strategies to reduce the basic reproduction rate of the disease (also known as R_0_) in different areas of the globe.

The relative success of these NPIs was demonstrated by their efficacy in controlling COVID-19 [4,5,6]. The deployment of NPI measures helped to delay the massive growth in cases in nations where such policies were adopted. The use of face masks, along with social-distancing measures, were the first control strategies to restrain SARS-CoV-2 transmission [7,8]. In fact, these fast, standardized, and cost-effective countermeasures were our only line of defense against the spread of COVID-19 incidences until the use of vaccines was approved [9].

Different studies have shown the positive effect of social distancing and the restriction of human mobility in controlling the spread of SARS-CoV-2 [10,11,12]. However, NPIs such as social distancing presented a major impact on poverty and inequality [13]. The epidemiological value of such interventions was less evident in lower-income countries, as they imposed a heavy burden on vulnerable groups [14,15]. Another important aspect of NPIs and COVID-19 itself is the toll on mental health that may have affected the perception of risk as well as the importance of individual and collective control measures [16,17,18,19]. Therefore, the investigation of the potential outcomes related to the full and partial implementation of NPIs is of paramount relevance for COVID-19 epidemiology.

In Brazil, the main reason for the adoption of NPIs was the increasing number of cases in 2020–2021, especially in settings where no measures to avoid crowding were implemented [20]. At that time, multiple cities suffered from an insufficiency of ICU beds and a shortage of healthcare workers and supplies, which elevated the number of cases and deaths [21,22]. In such a scenario, cities with a smaller gross domestic product and modest health equipment structures struggled to manage the increase in healthcare demand due to SARS-CoV-2 circulation. The failure of the country to implement effective control measures, the denial of the pandemic’s severity, and the use of inefficient early COVID treatments had a direct impact on the number of cases [23].

During 2020 and 2021, Brazil was under the government of the far-right president Jair Bolsonaro, whose administration advertised non-evidence-based pharmacological interventions and disregarded dependable public health measures such as the use of face masks and social distancing [23]. These actions also impaired the implementation of public health services and policies at the federal level and in other spheres of administration. During this period, the government made concerted efforts to demobilize analytical skills as a crucial component of the National Healthcare System [24]. Such strategies exposed, in particular, the somewhat unexpected size of the political bias regarding the spread of SARS-CoV-2 and the mortality rate of COVID-19 in Brazil, surpassing other important epidemiological elements such as the level of poverty and the mutation of the virus. People were constantly persuaded not to adopt restrictive measures [25,26]. Furthermore, the fomentation of anti-COVID-19 measures by the federal government amplified the challenges of monitoring the spread of the virus, to the extent that the daily count of cases and deaths was not properly updated. Such coordinated actions influenced decision-making processes in several other spheres of administration. Consequently, health managers were unaware of the true scope of the epidemic, which may have hindered the deployment of control measures at key points in the spread of SARS-CoV-2 [27].

States and cities run by political opponents of president Jair Bolsonaro deployed NPI measures independently of federal sanitary orientations [28]. An example was the city of Araraquara, São Paulo, Brazil. This city has an estimated population of approximately 238,000 people and a demographic density of 235 inhabitants/km^2^, according to IBGE [29]. Local authorities implemented different degrees of restrictive NPI measures, the red, yellow, and green flags, in response to the severity of the epidemiological scenario. Araraquara deployed strict social-distancing and lockdown measures to avoid the collapse of the health system as the cases of COVID-19 started to increase. The so-called red-flag measures limited certain economic activities and sectors but did not promote the complete shutdown of services, which happened when lockdown measures were implemented. Araraquara also issued two lockdown orders in 2021, one in February and another in June. As a result, there was a 43.02% decrease in cases after the first ten days of these interventions. Additionally, the time frame comprising March and June of 2021 presented more cases than reported in the previous 10 months [30]. As one of the largest cities in the region, Araraquara stood out nationally for implementing complete lockdown strategies and carrying out mass testing for COVID-19, which culminated in a decrease in death rates associated with the implementation of vaccination strategies in 2021 [31].

The diversity of the epidemiological patterns of COVID-19 during the pandemic was a result of the circulation of different SARS-CoV-2 variants [32]. Thus, it is important to evaluate the evolution of common symptoms; the influence of environmental indicators such as temperature, humidity, and air quality on the spread of the virus; the effect of events such as vaccination and crowding; sociopolitical actions in neighborhoods or cities; and the type of measures implemented to control the spread of the virus, which are among the factors whose patterns change throughout pandemics, seasonally or non-seasonally.

In order to identify and understand some of these disease patterns, researchers have demonstrated the association of COVID-19 cases with crowding and NPI implementation [8,9,33,34,35]. In this sense, COVID-19’s spread is regarded as a multifaceted sociopolitical problem, for which exploratory analysis and data comparison based on urban mobility and sociodemographic indicators can provide useful epidemiological information [36,37,38,39,40].

Hence, in order to better understand the pandemic scenario in Araraquara and what the forecasting of COVID-19, we applied mathematical and epidemiological models in neural networks to forecast the number of cases and observe the possible effects of restrictive measures in Araraquara, Brazil. Our assessment was based on previous studies that also used urban mobility and temporal and quantitative epidemiological data. Furthermore, by consulting the datasets thoroughly, we outlined the COVID-19 epidemiological scenario in Araraquara in 2020 and 2021, as well as the community mobility and its potential contribution to disease transmission. In addition, we used an initial Fourier transformation application to identify the disease’s seasonal spread despite the implementation of control measures, which demonstrated that there were seasonal events to which specific NPIs could have been applied in certain key periods.

## 2. Materials and Methods

In this section of the manuscript, we provide a comprehensive description of the datasets and methods used for the analysis. We also highlight important hallmark dates that may have influenced the epidemiological patterns of COVID-19 in Araraquara.

### 2.1. Dataset

In order to better comprehend the phenomena, we compared information from three different data sources:

- Community mobility reports (CMRs) from Google to analyze social dynamics;
- Municipal daily reports (MDRs) from bulletins provided by local authorities;
- Health department reports (HDRs) from local health authorities.

The CMRs [41] comprise data collected from tracked mobile phones/smartphones. The entries are associated with the activities and mobility of users in different sites and environments such as work, residential neighborhoods, and public areas. The CMRs are divided into six categories: retail and recreation, parks, groceries and pharmacies, workplaces, public transit, and residential. We collected mobility entries from February 2020 to June 2021. The CMRs include different levels of geographical organization, such as country, state, and city. The analysis of this dataset provided inputs of community mobility dynamics and local incidences during the red-flag and lockdown periods in Araraquara.

The COVID-19 epidemiological data present in the MDR dataset were obtained from daily bulletins provided by the local government [42]. This dataset is organized as a time series of cases, with the first registry dated 3 March 2020, and all subsequent data added daily. The dataset includes information such as the date of the report, Brazilian province, city, number of cases, number of deaths, estimated population, city code, percentage of cases per 100,000 inhabitants, and death rate.

The Health Department of Araraquara maintained a weekly updated database (HDR dataset) that was partially used in our analysis. The dataset comprised reports of 77,809 RT-PCR and rapid tests, including antibody or antigen test, and additional variables collected from 1 April 2020 to 31 May 2021, and was composed of 26 columns, organized as follows: identification column; three columns containing report date, date of onset of symptoms, and type and date of diagnostic test; one column related to the test result; one column identifying whether the user was a health professional; and other columns related to the patient’s chart, such as possible diseases and symptoms presented (see Table 1).

Finally, we considered important dates for our analyses, as organized in Table 2. The first dates were the national elections, which occurred on 15 and 29 November 2020, resulting in crowding. Additionally, Araraquara’s government implemented NPIs (red-flag alerts) during 2020 on 23 March, 1 May, 4 September, and 22 December. They also implemented NPIs on 2 April 2021. Further, they declared a lockdown on 21 February 2021, spanning to 2 March, and from 20 June to 27 June. Some of these dates explain the behavior of the temporal trends in Araraquara, as will be shown below.

### 2.2. t-Distributed Stochastic Neighbor Embedding (t-SNE)

The community mobility reports (CMRs) dataset provided a complex arrangement of mobility that evolved through many community sectors and changed over time when local events occurred. To generate independent variables capable of generalizing a temporal event or circumstance and simplify the data interpretation and visualization by reducing dimensionality through projecting the data to a lower-dimensional domain and preserving local structures, we used the t-Distributed Stochastic Neighbor Embedding (t-SNE) method proposed by Van der Maaten and Hinton [43], based on Stochastic Neighbor Embedding (SNE) [44]. However, there was a trade-off between precision (achieved through high dimensionality) and interpretability (given low-throw dimensions).

We identified works that used different methods for this task, such as those that used the PCA method, including the approach presented by Aragão et al. [34]. We adopted t-SNE, however, because it could take a high-dimensional dataset and reduce it to a low-dimensional graph while retaining the original information and meaning in the data. PCA (Principal Component Analysis) is a method for reducing the number of variables (generating principal components—PCs) in order to generalize the projections without losing substantial information [45]. To set up the sorting PC, the algorithm measures the average value of all dimensions of the data to avoid an unequal contribution from a given dimension into the process and applies a standardizing method to generate an input in a standard scale. Afterwards, the covariance of the variables is computed, and the covariance matrix ***A*** is established, followed by the use of a linear transformation to find the characteristic vectors (also known as *eigenvectors*). The chosen PC variables are the *eigenvectors* with the highest *eigenvalues*. The results are presented using an orthogonal axis designed to explain the maximum amount of variance in the set, which may result in information loss.

The t-SNE method worked by first calculating the Euclidean distances between points on the dataset, which were then translated into conditional probabilities that reflect the similarity between each pair of sites using:

(1) $$p_{i|j}=\frac{exp(\frac{-|x_{i}-x_{j}{|}^{2}}{2\sigma_{i}^{2}})}{\sum_{k\neq i}exp\left(\frac{-|x_{i}-x_{k}{|}^{2}}{2\sigma_{i}^{2}}\right)}$$

in which the division represents each possible cluster with different densities.

The next step was to create a random dataset in low-dimensional space and compute a joint probability distribution based on k-features (our target, in general, was 2 dimensions as the outcome). We established a joint probability distribution for this set of points, but at this stage utilized the t-distribution rather than the Gaussian distribution, as was the case in the initial dataset, adopting:

(2) $$q_{i|j}=\frac{\left(1+\right|y_{i}-y_{j}{{|}^{2})}^{-1}}{\sum_{k\neq l}\left(1+\right|y_{k}-y_{l}{{|}^{2})}^{-1}}$$

Finally, we used the Kullback–Leiber (KL) divergence measure to get as near to the original dataset’s joint probability distribution of data points in the low-dimensional space as possible. The value of the KL divergence decreases as the distributions become more similar, eventually reaching 0 when the distributions are identical. The KL divergence is defined as:

(3) $$D_{KL}\left(P|Q\right)=\sum_{x\in X}P\left(x\right)log\left(\frac{P(x)}{Q(x)}\right)$$

In the approach presented in this work, we applied this method to the CMR dataset. However, we removed the component that represented *residential* mobility, keeping only those components relating to the different interactions across the city.

### 2.3. Fast Fourier Transform—FFT

Certain events occur in a seasonal manner, and COVID-19 is influenced by seasonal events. Two examples are the temperature and humidity indexes, which are seasonal and have a relation to the dynamics of COVID-19 [33,46,47]. The Fourier transform (FT) may help us comprehend the construction of the COVID-19 curve using an alternative method.

First, a periodic signal can be represented by a Fourier series, which is an infinite sum of sinusoidal functions (cosine and sine), each with a frequency that is an integer multiple of *f* = 1/*T*:

(4) $$f\left(t\right)=a_{0}+\sum_{m=1}^{\infty }a_{m}cos^{\frac{2\pi mt}{T}}+\sum_{n=1}^{\infty }b_{n}sin^{\frac{2\pi nt}{T}}$$

where $a_{0}$, $a_{m}$, and $b_{n}$ represent the Fourier series coefficients, which determine the relative weights for each of the sinusoids.

In order to depict a function as a continuous or integral superposition of complex exponential functions, we can then use the Fourier transform. A series can thus be represented in the frequency domain. To perform this method, we used decomposition, which converts (or extracts) the signal as a sum of other signals or as the sum of a series of *n*-sine waves represented as continuous signs.

In the case of non-continuous problems with information about a set of data rather than a continuous function, the discrete Fourier transform (DFT) could assist in transforming this dataset into an equal-sized set with information about the frequencies of the function that satisfies the dataset, or it could provide the discretized form of the Fourier transform. An arbitrary function that is periodic on a certain domain must be projected into orthogonal function directions by expanding it as a sum of sines and cosines as well as periods on that domain. For the 1-dimensional DFT (y[k]) of length N, we used the following definition:

(5) $$y\left[k\right]=\sum_{n=0}^{N-1}x\left[n\right]e^{-2\pi nk/N},k=0,\ldots ,N-1$$

where the daily number of SARS-CoV-2 cases reported on the $n_{th}$ day of the time series is represented by *x*, and y[k] denotes the magnitude of the $k_{th}$ frequency on the *n*-day of the time series. By converting the time series of the number of new daily SARS-CoV-2 cases to the frequency domain, we used the DFT to estimate the period’s length in the COVID-19 data spectrum.

To compute the discrete Fourier transform of a signal, a computer needs to complete N multiplications × N additions, resulting in an algorithm with a complexity of O(*N*^2^) operations. The fast Fourier transform (FFT), as the name indicates, is a method that finds the discrete Fourier transform of an input much quicker than computing it directly. The FFT algorithm decreases the number of calculations required for a problem of size N from O(*N*^2^) to O(*NlogN*), using the so-called *divide-and-conquer* approach. Rather than working with big signals, the algorithm breaks them into smaller ones and applies the DFT to these, before summing all of the smaller DFT results to obtain the final outcome, asymptotically providing a significant advantage. Hence, the FFT is an algorithm that allows one to quickly transform a series of discrete signals into a sample containing the frequencies of these signals, as long as certain properties are met.

Given this advantage, the intent was to consider the time series of COVID-19 cases extracted from the MDR dataset. The FFT allowed us to extract and map event frequencies for the city of Araraquara, displaying seasonality throughout the year. The frequencies were determined by the function and reported in a graphical form using four categories: (1) those representing events with seasonality ranging from 9 to 12 months (40–52 weeks); (2) those representing events with seasonality ranging from 6 to 9 months (26–40 weeks); (3) those representing events with seasonality ranging from 3 to 6 months (12–26 weeks); and (4) those representing events with seasonality ranging from 1 week to 3 months (1–12 weeks).

If a frequency peak appeared in a specific sector, such as between 3 and 6 months, it meant that the city had a high rate of COVID-19 cases, which repeated itself in a 3–6 month period. This method may enable one to map the events that correspond to each seasonality frequency and identify the best times to implement a measure, in addition to understanding its magnitude in the context of case control reporting.

### 2.4. Seasonal Auto-Regressive Integrated Moving Average—SARIMA

The ARIMA and SARIMA methods are used in several applications, especially in univariate tasks. It is possible to refer to many works that used these methods to perform regression during the COVID-19 pandemic [34,48,49,50,51,52]. The methods consist of combining auto-regressive (AR) and moving-average models (MA) and then defining the *ARIMA*(*p*,*d*,*q*) model, allowing one to accommodate different types of non-stationary time series (*d* is the order of differentiation applied for the time series).

*AR*(*p*) is a *p*-order auto-regressive model that is based on a linear combination of past observations $X_{t}=\alpha_{1}X_{t-1}+\alpha_{2}X_{t-2}+...+\alpha_{p}X_{t-p}+\varepsilon_{t}$, where α is constant and $\varepsilon_{t}$ is a stochastic disturbance from the white noise process. When adjusting the parameters of an auto-regressive model, the parameter $\alpha_{k}$ must be estimated, resulting in the equation:

(6) $$Y_{t}=\mu +X_{t}=\mu +\alpha_{1}X_{t-1}+\ldots +\alpha_{p}X_{t-p}+\varepsilon_{t}$$

On the other hand, MA models are an extension of the white noise process, consisting of a linear combination of the disturbance $\varepsilon_{t}$ added to recent disturbances $\varepsilon_{t-1},\varepsilon_{t-2},\ldots ,\varepsilon_{t-q}$. Thus, a *q*-order MA model can be defined as follows:

(7) $$X_{t}=\varepsilon_{t}+\beta_{1}\varepsilon_{t-1}+\beta_{2}\varepsilon_{t-2}+\ldots +\beta_{q}\varepsilon_{t-q}$$

Seasonal Auto-regressive Integrated Moving Average (SARIMA), also known as Seasonal ARIMA, was created to support univariate time series based on ARIMA models. It contains a second set of parameters (*P*,*Q*,*D*)_*s*_ referring to seasonal auto-regression in the seasonal period *s*.

The advantage of using the SARIMA model is that there are fewer hyper-parameters, making the configuration file readily manageable if the model is put into production. However, when *p* and *q* rise in value, there are more coefficients to fit, increasing the time complexity exponentially. If *p* and *q* are large, the procedure becomes difficult to fit, depending on the complexity of the dataset.

### 2.5. Transformers

Certain methods are useful for situations that cannot be addressed linearly. In such circumstances, neural networks allow one to detect the degree of links among variables, such as those in our study, because non-linear variables cannot be described. Hence, neural networks show promise for univariate, bivariate, and multivariate regression issues.

One possible approach is to use the Transformer Multi-Head Attention model (or simply the Transformer model), which excels at managing sequential data, allowing one to tackle sequential and temporal topics without being limited in prediction. The core of the *Transformer* model’s architecture is based on attention mechanisms [53]. Transformers have demonstrated outstanding performance in natural language processing and (especially) in computer vision, which the scientific community has exploited in the time-series domain [54]. This method has the advantage of capturing long-term data dependencies and relationships and producing better data visualizations.

The Transformer model’s outermost structure consists of an encoder and a decoder, and the core is built on self-attention method processes. When adding positional encoding to the input and output sequence embedding, self-attention allows the model to search at other points in the input sequence for hints that might assist in better encoding a target sequence. This approach uses positional encoding appended to input embedding to describe the input series representation, with no recurrence or convolution operations. The inputs to both the encoder and decoder components use the same embedding and positional encoding logic, taking both sequences of input and output tokens and converting them into vectors with no order (a query vector, key vector, and value vector for each input). Afterwards, a component of the embedding vectors called the positional encoding converts the sequence to sine and cosine frequencies. In this case, nearby elements have a similar positional encoding.

Each encoder block has a multi-head self-attention module and a position-wise feed-forward network (FFN), while each decoder block inserts cross-attention models between the multi-head self-attention module and the position-wise feed-forward network. The multi-head attention mechanism uses the values, keys, and queries in the so-called Scaled Dot-Product Attention process to calculate the attention weights based on:

(8) $$Attention\left(Q,V,K\right)=softmax\left(\frac{QK^{T}}{\sqrt{d_{K}}}\right)V$$

where *Q*, *K*, and *V* are *queries*, *keys*, and *values*, respectively, and $d_{K}$ is the dimension key, which are used to regularize the result with a softmax function. The softmax function layer then turns these scores into probabilities (all positive, adding up to 1.0). The cell with the highest probability is chosen, and the input associated with it is identified as the output of the time step.

The network adopted in this work had a head size of 200 blocks, with 4 heads containing transformer blocks connected to an MLP with 128 units and a dropout of 30%. We used the univariate model based on COVID-19 daily cases as the input data to predict two different outcomes: the next day following a true sequence, and the next day following a sequence that was constructed based on each sequential prediction. The learning algorithm was asked to output the function f:R→R in order to complete this task.

## 3. Experiments and Results

We started by extracting information based on graphs that were constructed for data from the MDR, HDR, and CMR datasets during 2020 and 2021.

### 3.1. COVID-19 Patterns in Araraquara

From the HDR dataset, we identified 77,809 records, and the overall sum of positive tests (including RT-PCR and fast tests) was 14,729; tests that presented negative to COVID-19 comprised 47,432 records. Additionally, 52 sample records were designated as inconclusive or uncertain. From the 14,729 COVID-19-positive tests, 1838 (approximately 12.5%) were reported as individuals with symptoms, whereas 12,891 (approximately 87.5%) were recognized as COVID-19-positive individuals who were asymptomatic and did not report symptoms.

**Figure 1:**
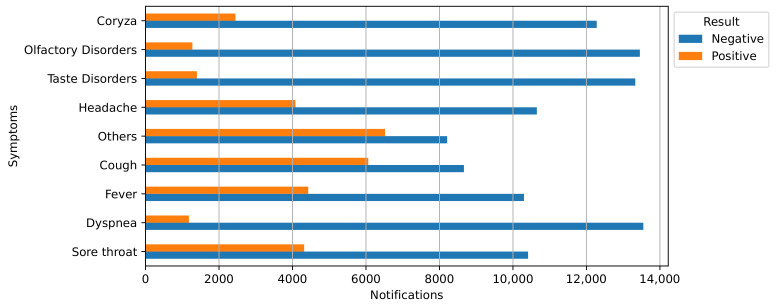
Symptoms reported by suspected (blue) and confirmed (orange) COVID-19 patients from Araraquara, São Paulo, Brazil, from March 2020 to July 2021.

The most frequently reported symptoms among the positive-RT-PCR patients were cough (reported by 41.16%), fever (30.06%), sore throat (29.30%), and headaches (27.69%), as shown in Figure 1. Additionally, we observed that when users were identified as positive, the symptoms related to olfactory, gustatory, and dyspnea disorders were the least reported.

**Figure 2:**
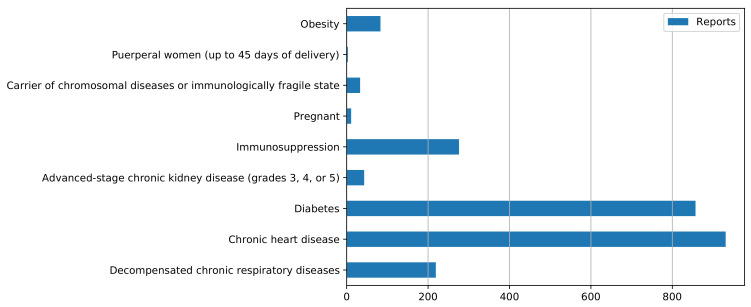
Types of comorbidities and health conditions presented by residents of Araraquara, São Paulo, Brazil, comprising cases reported from March 2020 to July 2021.

Among those individuals identified as positive cases, 2456 had some condition or comorbidity that characterized them as a special-attention case or was considered a risk factor (Figure 2). This group was distributed under the following classifications according to the individual’s condition: obesity; postpartum or pregnant women; patients with chronic respiratory, cardiac, renal, or chromosomal diseases or frailty; and patients with diabetes or immunosuppression.

In relation to the importing and exporting of cases, the movement of the disease between cities within the state of São Paulo and its neighboring states had a significant influence. For example, 7888 out of the 77,809 case records identified as Araraquara residents were imported from cities in other states, such as Curitiba, located in the neighboring Paraná state. The distance between Araraquara and Curitiba is approximately 664.3 km.

**Figure 3:**
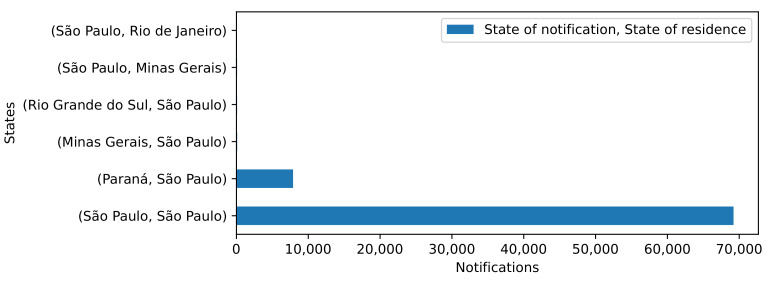
Autochthonous and imported reported cases, state of São Paulo, Brazil, 2020–2021.

The state of São Paulo borders four other states: Rio de Janeiro, Minas Gerais, Paraná, and Mato Grosso do Sul. Figure 3 depicts the cases imported and exported to/from other states, including Minas Gerais and Rio de Janeiro. These three states are the most populated states in Brazil according to the IBGE [29], with São Paulo representing 21.9% of the Brazilian population, Minas Gerais 10.1%, and Rio de Janeiro 8.2%. Considering the states with more than 80 instances imported or exported to another state, the emigration from Paraná to São Paulo represented 7664 records, and that from Minas Gerais and Rio Grande do Sul to São Paulo accounted for 104 and 94, respectively. Emigration from São Paulo to Minas Gerais accounted for 89 of the instances. The remaining cases for São Paulo originated in the state itself (69,442 cases).

### 3.2. Community Mobility and Frequency Events

**Figure 4:**
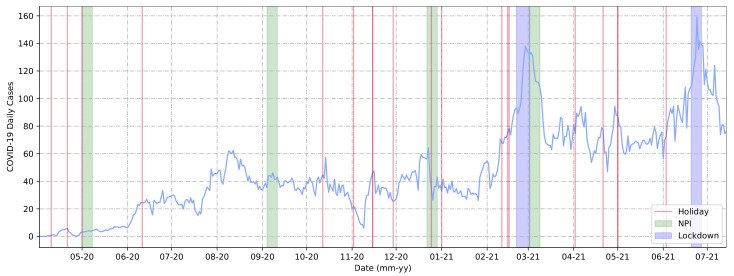
Cases reported (blue line) by health authorities from Araraquara, São Paulo, Brazil, from March 2020 to July 2021.

It was possible to extract event-based information for 2020 and 2021, in particular, in relation to the variation in COVID-19 daily cases. To this end, we used the MDR dataset that is graphically represented in Figure 4. In this graph, for ease of understanding, we applied a moving average of 7 days and included all official public holidays in Brazil as red lines. Additionally, the events presented in Table 2 (Section 2) were included, i.e., national elections, NPIs, and lockdown measure implementations.

Lockdown periods are represented as blue stripes, indicating the exact length in relation to the timeline and the determined period span. Green stripes correspond to NPI measures, representing the first day of the measure’s implementation plus the seven days following. We adopted this approach as it could make it easier to observe how these measures graphically demonstrated their outcomes in the period of 7 days. As shown in Figure 4, these types of measures, such as NPIs and lockdowns, were implemented when the curve’s trend was at its local peak (when considering a short period, this could be determined as the peak of cases in the selected period).

There was a local peak between August and September 2020. During this period, the government of Araraquara authorized bars and restaurants to operate, which may have contributed to the increasing number of cases. Given the increasing number of cases during other periods, we assumed that the purpose of implementing these measures was to cause a recession or spread stagnation. Furthermore, we observed that at least half of the holiday dates appeared to precede an increase in daily cases, contributing to the upward trend of the curve. This was visible during periods such as the holidays preceding the March 2021 lockdown (03-21 in the graph). Some holidays in 2021 followed the same pattern, such as those in April and June (04-21 and 06-21 in the graph). It is important to note that the lockdown periods were implemented immediately after the highest rates of COVID-19 daily cases in Araraquara.

**Figure 5:**
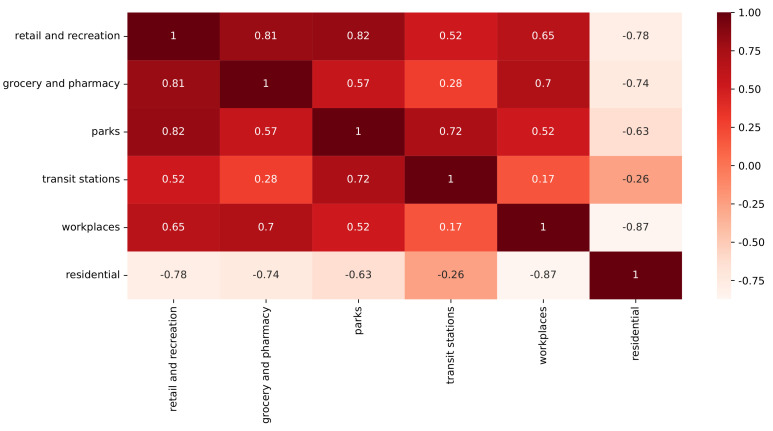
CMR feature correlation.

We used data from the CMR database and ran them through the t-SNE method to help us understand how these high numbers of daily COVID-19 cases came to be. However, first, we verified if there was a correlation between the CMR features (Figure 5). With the exception of transit stations, residential mobility had an inverse connection with all other components. The other elements were positively connected to one another. Furthermore, the strongest negative correlation was between the residential and workplace features.

**Figure 6:**
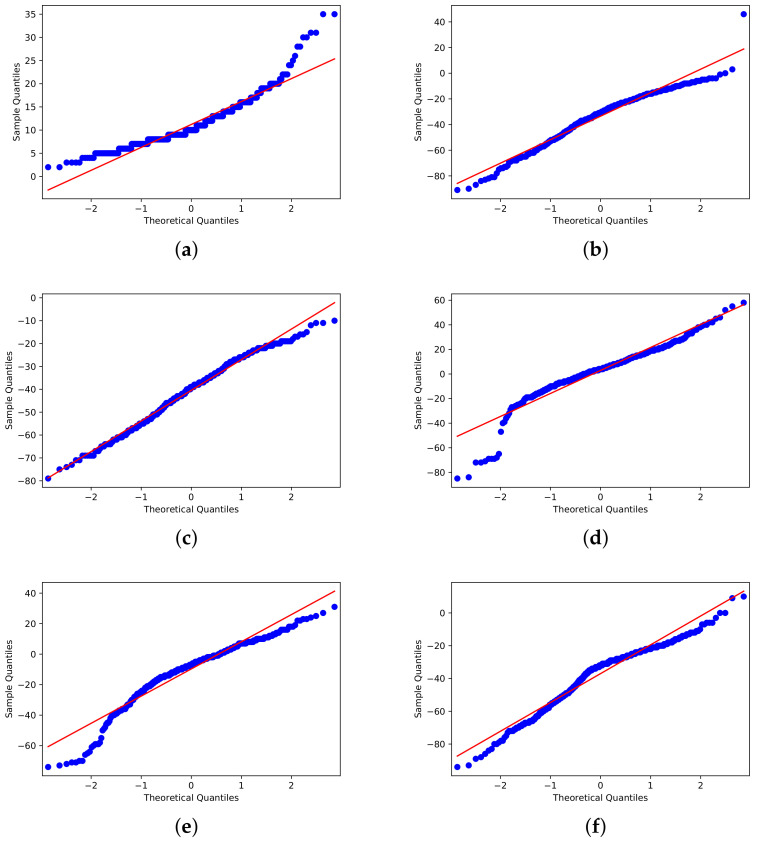
Shapiro–Wilk outcomes: (**a**) Residential. (**b**) Parks. (**c**) Transit Stations. (**d**) Groceries and pharmacies. (**e**) Workplaces. (**f**) Retail and Recreation.

Furthermore, before employing t-SNE, we conducted a Shapiro–Wilk test [55] to see if the distributions were close to normal distributions (Figure 6). All CMR characteristics had *p*-values equal to 0.000, leading us to reject the null hypothesis $H_{0}$ (that the data would follow a pattern of development) and indicating that the data did not display Gaussian distribution behavior.

**Figure 7:**
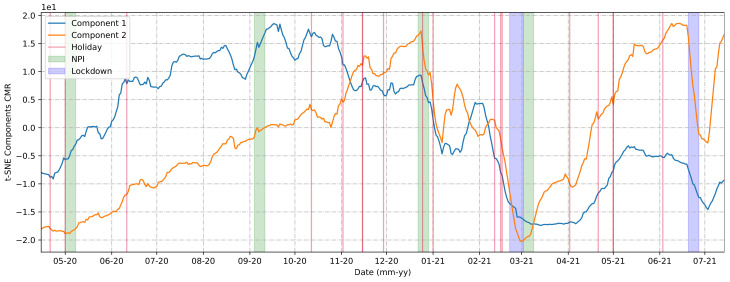
t-SNE Components.

The t-SNE method could help us reduce the number of community mobility components analyzed from five (retail and recreation, parks, groceries and pharmacies, workplaces, and public transit) to two, as shown in Figure 7. The algorithm looked for similarities between all the points based on their distance and divided them into two groups, which we will refer to as Component 1 and Component 2.

Comparing the components generated by the t-SNE method and the holidays, NPIs, and lockdown measure implementations, we could determine that:

- The first NPI was adopted one month before the first wave of cases in the city.
- The second and third NPIs were implemented at a time of a high and stable number of cases.
- The mobility behaviors presented in the first and second lockdowns were not similar. The first lockdown demonstrated a greater effective mobility restraint force compared to the second.
- Comparing with Figure 4, it was observed that the mobility restrictions could reduce the number of cases but were not so efficient as to decrease the number of daily cases to zero.
- The rises in the curves of the urban mobility components, in general, were preceded by a holiday.

We then applied the FFT in an attempt to specify if there were periods during which events were distributed that influenced the case curve. It is important to note that in this case, the FFT method was only used to identify the Fourier frequency spectrum, and the method was applied to the original temporal record of cases, i.e., the series was applied without using a moving average.

**Figure 8:**
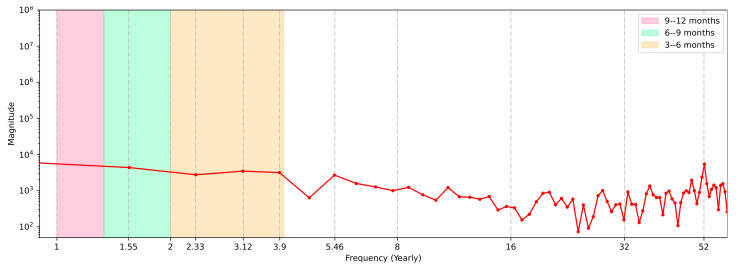
COVID-19 FFT frequency events.

Figure 8 depicts a frequency spectrum with endpoints at junctions that correspond to COVID-19’s case peaks. It was necessary to convert this to a semi-logarithmic annual scale, in which each peak is represented by its magnitude (on the *y*-axis) and *k*-frequency (on the *x*-axis). Without the 7-day moving average treatment, the DFT was applied to the original curve.

The graph shows a frequency peak for the 7-day cycle associated with the case notification process (with accumulated cases reported on Sunday and Monday), which was repeated 52 times a year (k=52), where 52 is the number of weeks in a year. Furthermore, we discovered another point with a high magnitude on the extreme left side of the spectrum that was not categorized as a peak, which was a phenomenon that occurred every 6 to 9 moths of the year during the observation period. This implied that the repeat cycle was roughly *k* = 1.55. This phenomenon could be identified as the presence of so-called popular waves, defined as a sudden increase in the number of cases compared to the city’s average.

Furthermore, three other phenomena or events recurred over a period of 3 to 6 months (k=[2.33,3.12,3.9]). Except for the event at k=52, we could identify this as a single moment that appeared to be repeated every 1 to 3 months, that is, with *k* = 5.46.

### 3.3. Forecasts

In the following, we present some predictions that could support and help in determining if it is possible to introduce the hypothesis that lockdowns were an important measure to contain or reduce the COVID-19 daily cases in Araraquara.

#### 3.3.1. SARIMA

**Figure 9:**
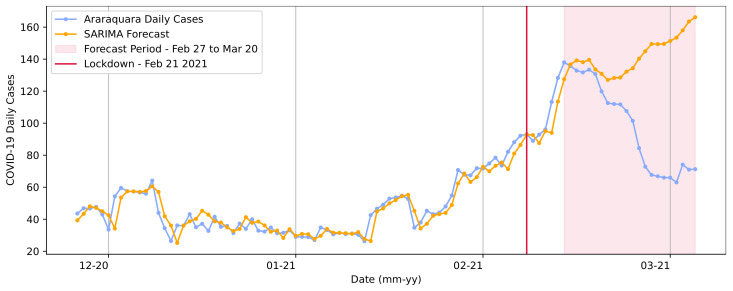
SARIMA model forecasts for the first lockdown from 21 February to 2 March in Araraquara.

The first regression approach for the two lockdown measure periods used the SARIMA model. In the training step, it was observed that the model maintained the curve’s trend and its patterns when compared to the ground truth throughout the predictions, as shown in Figure 9.

Figure 9 presents the results from a trained SARIMA model. The base data for the training step ranged from the first reported case (25 February 2020) to the day of the first lockdown (21 February 2021). The model parameters—included in the definition $SARIMA\left(p,d,q\right){\left(P,D,Q\right)}_{s}$—were determined using the Box and Jenkins approach [56,57]. The model applied for the first lockdown period was defined in the order (0, 1, 0), with the seasonality order (7, 1, 0, 7).

We also considered that an infected person’s symptoms begin to appear approximately 4 or 5 days after infection, as presented by Johansson et al. [58]; hence, if the transmission took place on day *d*, we could observe its results on day d+7 [1,59]. Given this, when choosing the period for prediction (the data used in the test), we considered that results could occur within a timespan of 6 days after the first day of lockdown implementation, representing the future outcome of the implemented measures in terms of COVID-19 daily cases.

The predictions made by the model in the test step demonstrated that it was expected that the curve’s trend and the number of cases would increase. The real number of cases in 20 March 2021 was 71. For the same date, the model predicted a value of 166.17, representing a value difference of approximately 94 cases. Most importantly, the RMSE extracted based on the sweeping curve was RMSE = 4.55 cases.

**Figure 10:**
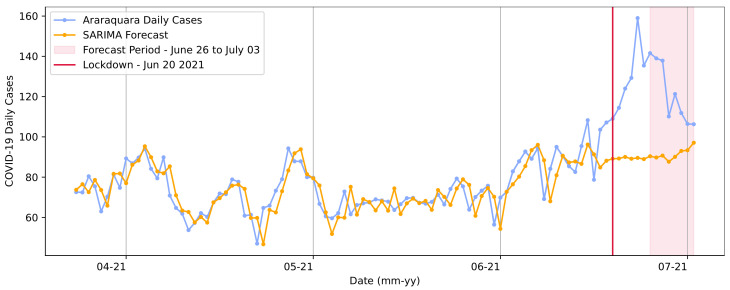
SARIMA model forecasts for the second lockdown from 20 to 27 June in Araraquara.

Furthermore, the model was trained for the second lockdown period and defined in the order (0, 1, 0), with the seasonality order (8, 1, 0, 7). The predictions for the second lockdown are shown in Figure 10.

We observed different patterns for the curve’s trend and different COVID-19 daily case expectations in this prediction set. In contrast to the first lockdown, the forecasts showed that the number of cases would remain stable, with no significant increases. This result may have been based on the previous data pattern curve, which was presented above for the beginning of that timespan.

The real number of cases in 3 June 2021 was 106. The model predicted a value of 97.09 for the same date, representing a value difference of approximately 9 cases. However, the model showed a significant value divergence for previous dates in that period. The RMSE extracted based on the complete curve was RMSE = 5.57 cases.

#### 3.3.2. Transformer Model

Next, we present the predictions made using the Transformer models. We decided that, in order to predict one day, a sequence of seven days had to be presented, which meant that in order to predict the eighth day, we had to first present data corresponding to seven consecutive days of COVID-19 cases. The model received a sequence of data in both the training and testing stages, forming a vector of information. Similarly, to predict day 9, we presented a 7-day sequence that corresponded to days 2–8. To forecast day 10, we presented data from days 3 to 9.

**Figure 11:**
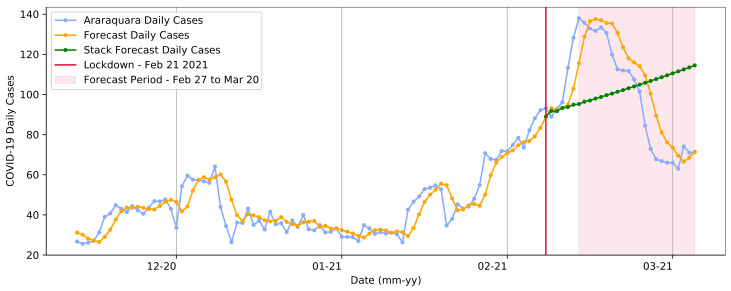
Transformer model forecasts for the first lockdown from 21 February to 2 March in Araraquara.

Figure 11 depicts the actual COVID-19 daily cases, those predicted by the model for the same timeline, and the forecast for cases immediately after the lockdown was implemented on 21 February 2021. This last predicted series was obtained with the assumption that the forecast day should be included in the following time series used with the forecast. In this case, suppose we use the real series from days 1 to 7 to forecast the next day (eight days): $\left[1_{d},2_{d}\ldots 6_{d},7_{d}\right]\to 8_{p}$. Once the forecast for day 8 is complete, its value is attached to the next series as a data entry, which means that for day nine, we consider days 2 through 7 plus the previously predicted eighth day: $\left[2_{d},3_{d}\ldots 7_{r},8_{p}\right]\to 9_{p}$.

As observed, the pattern learned by the model demonstrated that the predicted data expected that the trend in the number of cases would continue to rise, although not as sharply as the current data presented. This could be explained by hidden information that was not presented, such as the fact that crowding had occurred in the previous period, which could be critical for the model to determine a trend more effectively and accurately. As with the SARIMA model’s predictions, once real temporal information was not provided, the model predicted an increase in the number of cases due to COVID-19. At the test stage, the model had an RMSE of 13.30. The sum of COVID-19 cases during the test period was 2783, while the sum of values predicted by the model for the same time period was 2878.

**Figure 12:**
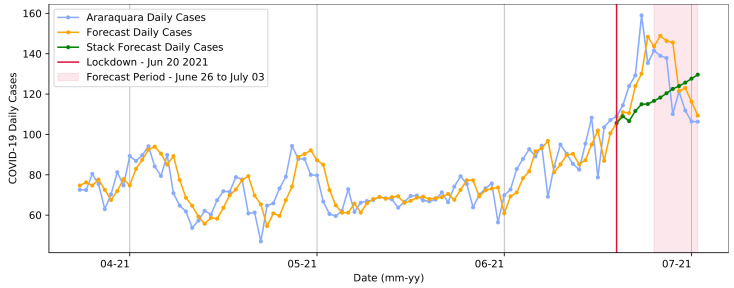
Transformer model forecasts for the second lockdown from 20 to 27 June in Araraquara.

Similarly, Figure 12 demonstrates the same pattern in predicting the days that occurred after the first lockdown. In this second timespan, the pattern diverged from the predictions made by the SARIMA model. This showed that the restriction measure’s implementation had a significant impact, preventing the recognized standard expectation of an increase in cases due to the oversaturation of the health system. In this process, the model presented a slightly higher RMSE than in the previous stage, with a value of 14.39 in the test step. For the test data, the total number of COVID-19 cases was 1746, whereas the sum amount predicted by the model was 1745.

## 4. Discussion

In the present study, we were able to assess the epidemiological profile of COVID-19 in a medium-sized city in Brazil, applying mathematical models and neural networks to analyze disease reports, community mobility, and NPIs. The ability of the models to properly forecast disease patterns in Araraquara supported their use as potential algorithms for situation diagnosis in different epidemiological scenarios. Additionally, our data strongly suggested that lockdown measures were of paramount importance in decreasing COVID-19 incidences in the city after only a few days of implementation.

It is not possible to state that the only factor that influenced the curve of cases was the use of NPIs. However, we identified that the implementation of lockdowns was a crucial factor that was independent of other factors, as demonstrated in Figure 4, and showed that as the pandemic progressed, the city benefited from the drastic drop in the number of cases. Additionally, in terms of epidemiology, vaccination itself helped decrease cases independently of other strategies. More research will be carried out in the future to gain a more comprehensive understanding.

Autoregressive models have been widely used to predict infectious diseases, especially COVID-19 [60]. SARIMA models were accurate and reliable in predicting deaths in 12 countries that presented high incidences for COVID-19. Importantly, the models properly adapted to the implementation of data, despite seasonality and complex patterns [61]. SARIMA and ARIMA models were used to generate a 60-day forecast of cumulative COVID-19 cases for the top 16 countries. The models were country-specific and were able to classify three categories of case growth [62]; the autoregressive models properly predicted dengue infection [63], monkeypox disease [64], and cases of hemorrhagic fever with renal syndrome [65].

The changes in epidemiological patterns shown in our analysis varied as the pandemic progressed, which is expected in time-series assessments [66]. Thus, the evolution of control measures and the management of cases had to be correlated with the variability of the patterns exhibited in one urban setting. In the case of Araraquara, symptoms, for example, presented different patterns during our analysis. For instance, initially, the community and governors focused on symptoms related to olfactory and taste disorders [67,68,69,70,71], but as shown in Figure 1, it is possible to highlight that the symptoms most related to positive cases were fever, cough, and sore throat (and those described as *Others*). Furthermore, the relative rate of positive and asymptomatic cases showed that the silent spread of the disease once voluntary consent to be tested was provided frequently occurred after the symptoms were experienced. Understanding these pattern changes could also contribute to greater community adherence to undergoing mass testing programs.

The more aspects revealed, the more reasonable the disease perception and understanding were; however, this also demonstrated how complex it is to produce accurate forecasts due to multi-factor deliberations. Non-temporal external static variables could be considered; in other words, a location in a city, for example, is a static variable, because it has multiple inherent features that make it unique.

The Brazilian political, economical, and social scenarios influenced the deployment of public health policies at a time when SARS-CoV-2 spread was a complicated issue with multiple contributing factors [72] also occurred in Araraquara. Another important aspect is the anti-vaccine or anti-public-media movement [73,74,75], which is frequently fueled by influential misinformation [76]. Aside from these anti-social factors, various environmental and anthropogenic disaster contexts have emerged as being interconnected in the fight against COVID-19 [77].

To improve the comprehension of this problem, once implemented in Araraquara, a massive testing program could assist in counting and demonstrating the value of preventing under-reporting; understanding the origins of epidemics; and the ability to take immediate action to ensure the containment of infected people, whether they are symptomatic or not. The significant efficacy of detecting such profiles via an efficient surveillance system, both quantitatively and qualitatively, as well as case analysis, leads to a better understanding of disease dynamics.

The Brazilian cities and states that implemented lockdowns exhibited positive but varied results, and in general the lockdown decisions were made due to crowding and a lack of ICU beds, not as a measure to prevent disease spread [78,79], as demonstrated in Figure 4 and Figure 7 (Section 3.2). Despite the positive results, not all governments decided to implement this type of measure, which may also have contributed in numbers to their territorial neighbors who implemented lockdowns. Considering these factors, therefore, it is important to analyze the periodicity of case numbers and the mapping of key events, in addition to the need for mass tests. Approaches such as those shown in Figure 8 (Section 3.2) could help with this mapping.

These measures may aid in the reduction of forecasting noise. On the one hand, the model should be highly adaptive; on the other hand, time sequences can be fairly complex or noisy, while others can be modeled simply with naive seasonal predictors and output prediction intervals that reflect the prediction uncertainty. This justifies the mapping of events, as well as the mapping of events inducing crowding (holidays) [34] based on community mobility, and local climate changes. For example, the discussions conducted by Wu et al. [46] and by Aragão et al. [36] showed that weather indices and the spread of COVID-19 were related.

## 5. Conclusions

This work aimed to provide a better understanding of the COVID-19 scenario that occurred in the city of Araraquara during 2020-21, considering the import and export of reported cases and the main symptoms presented by the population. Furthermore, some intriguing questions arose from Figure 7, showing the two components produced by the t-SNE method, which simplified our understanding of urban mobility in the city. Considering these components, we observed that mobility measures could be important for certain contexts, such as holidays, to avoid a worse scenario. It was also observed that after the implementation of lockdowns, there was a drastic decrease in urban mobility rates, which contributed to the drop in the number of cases of COVID-19 after a few days, but the same behavior did not necessarily apply to other NPI implementations.

Different regression models such as SARIMA and the Transformer model could present different results but similar behaviors, that is, the expectation was an increasing number of COVID-19 cases until the moment the lockdown measure was implemented. Nonetheless, because the virus contagion problem was multi-factorial, this factor (the imposition of the lockdown measure) was only one of the possible variables of interest that could influence the dynamics of COVID-19. It was observed that once a lockdown was implemented, the number of cases decreased but did not reach zero, and that after the restrictive measure was lifted, the number of cases increased again, reaching a similar level to before the lockdown. This could be explained by the extreme contagion rate of COVID-19, which maintains its transmission from person to person even with a low mobility rate within the population. Those who travel were still able to maintain viral transmission. Subsequently, when the restrictive measures were lifted, the virus achieved full activity in the population again. We believe that this behavior will continue until the whole population is vaccinated or the number of infected people who cannot be re-infected becomes high enough.

Aside from producing significant findings indicating that we are on the correct track, our existing data were not definitive, and more efforts are needed to provide a full and final decision on the strengthening of forecasting models. To this end, future studies will need to rank elements, taking into consideration the literature’s agreement on the determinants of the COVID-19 infection rate, such as the work presented by Fermo et al. [80] identifying more than 50 possible factors. Additionally, it is possible to observe that the use of the FFT could be essential in the mapping of events over the year, helping decision-making governors fight COVID-19. The findings could help us catch seasonal patterns that repeat over a period of a year. Instead of implementing measures for several repeated outbreaks, this would help in planning and implementing measures at a specific frequency. Without a precisely specified periodicity, Fourier spectrum analysis might be useful for comprehending COVID-19 waves that return with cycles of varied lengths (1 year or less). Finally, using a large number of variables as the input may reduce the noise in predicting the case numbers and make the model independent of these COVID-19 case registries.

Identifying a social event related to crowding and the way the disease manifested in the infected group could aid in future control measures and help identify potential events where an NPI should be used. We would need to compare cities in the same region to understand if these containment measures varied in their ability to decrease incidences, which we intend to evaluate in future work.

## Figures and Tables

**Table 1:** Health department reports (HDRs) data columns.

Report number	Sore throat	Obesity
Report date	Dyspnea	Coryza
Date of onset of symptoms	Fever	Asymptomatic
Test date (PCR/rapid)	Cough	Race/color
Result (PCR/rapid)	Others	Pregnant
Olfactory disorders	Headache	Diabetes
Chronic heart disease	Taste disorders	Puerperium (up to 45 days after labor)
A healthcare professional?	Carrier of chromosomal diseases or immunologically fragile state	Advanced-stage chronic kidney disease (grades 3, 4, or 5)
Uncompensated chronic respiratory diseases		

**Table 2:** Calendar of main events in Araraquara.

National Elections	NPI	Lockdown
15 November 2020	23 March 2020	21 February 2021
29 November 2020	1 May 2020	20 June 2021
	4 September 2020	
	22 December 2020	
	2 February 2021	

## Data Availability

The code and data used in this research can be found at https://github.com/Natalnet/ncovid-araraquara-paper, accessed on 1 January 2023.

## Abbreviations

The following abbreviations are used in this manuscript:

CMR	Community mobility report
DFT	Discrete Fourier transform
FT	Fourier transform
FFT	Fast Fourier transform
HDR	Health department report
IBGE	Brazilian Institute of Geography and Statistics
KL	Kullback–Leiber divergence
MDR	Municipal daily report
MLP	Multilayer perceptron
NPI	Non-pharmaceutical intervention
RMSE	Root-mean-square error
RT-PCR	Real-time polymerase chain reaction
SARIMA	Seasonal Auto-regressive Integrated Moving Average
T-SNE	t-Distributed Stochastic Neighbor Embedding
